# A revised taxonomy of the ammonoid genus *Desmoceras* from Japan and southern Sakhalin

**Published:** 2004-05-01

**Authors:** Tatsuro Matsumoto, Tamio Nishida

**Affiliations:** *) c/o The Kyushu University Museum, 6-10-1, Hakozaki, Higashi-ku, Fukuoka 812-8581, Japan; **) Faculty of Culture and Education, Saga University, 1, Honjo-machi, Saga 840-8502, Japan

**Keywords:** Ammonoidea, *Desmoceras*, Cretaceous, Albian, Cenomanian, Turonian

## Abstract

Based on the stratigraphically well sorted material from Japan and southern Sakhalin, the ammonoid species belonging to the genus *Desmoceras* are classified as follows in ascending order: *D. latidorsatum* (Michelin) (Middle to mid-Upper Albian), *D. dawsoni shikokuense* (Yabe and Shimizu) (Upper Albian), *D. kossmati* Matsumoto (uppermost Albian–Lower Cenomanian), *D. japonicum* Yabe (uppermost Albian–Cenomanian), and *D. ezoanum* Matsumoto (mid-Upper Cenomanian–mid-Lower Turonian). *D. japonicum* can be subdivided into the earlier and later subspecies. *D. poronaicum* Yabe is a junior synonym of *D. japonicum*. Despite the scarcely ornamented shell, each taxon can be defined by its own characters.

## Introduction

**Fig. 1:**
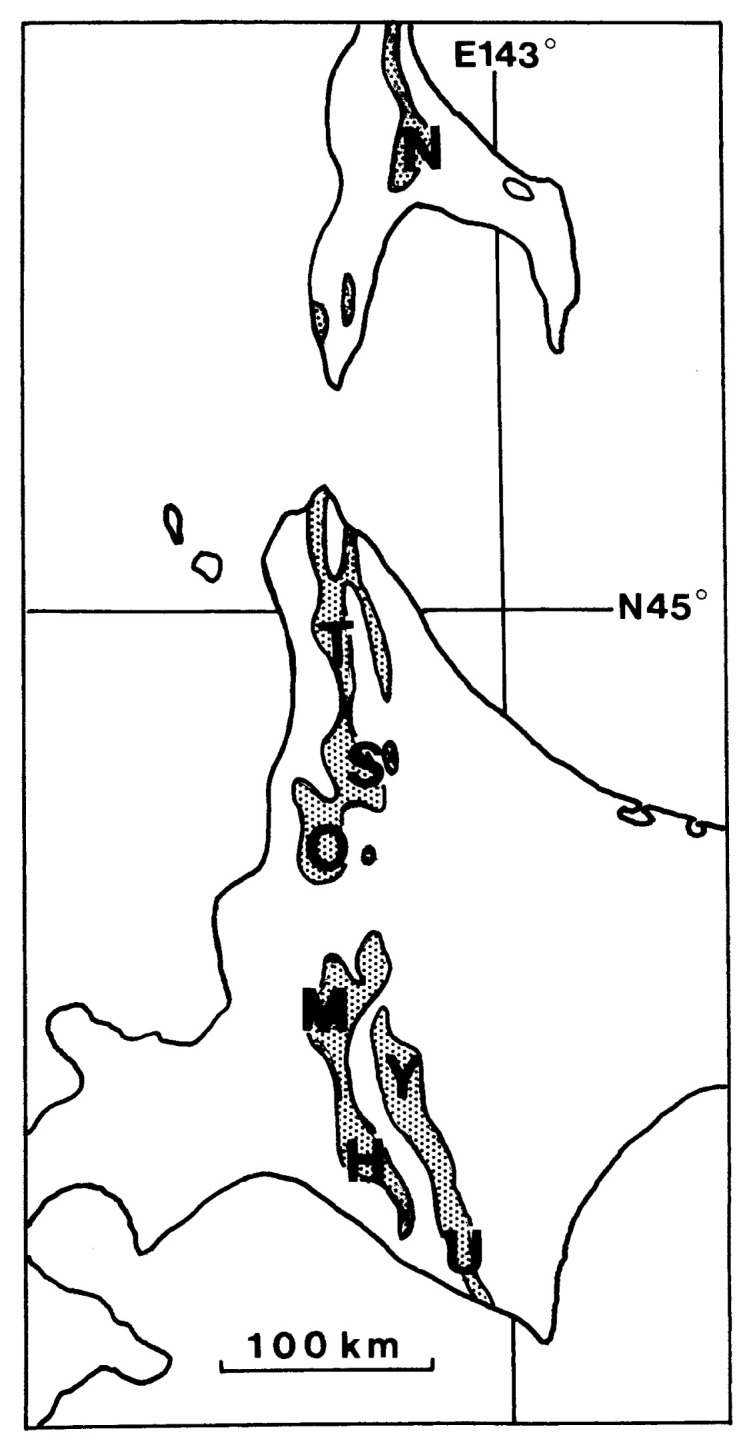
Map of southern Sakhalin and Hokkaido (main part), showing the distribution of the Yezo Group. The area where the mid-Cretaceous (Upper Albian–Lower Turonian) series has been intensively studied are indicated; from north to south: N = Naiba [Naibuchi], T = Teshio Nakagawa, S = Soeushinai, O = Obirashibe valley, M = Mikasa and vicinities, Y = Yubari Mountains (Shuyubari or Oyubari), H = Hobetsu, U = Urakawa and vicinity.

Since the pioneer work of Yabe,1) several species of the genus *Desmoceras* Zittel, 1885 2) have been described from Japan and southern Sakhalin (see Fig. 1). Recently we have restudied the available material and noticed some points to be revised. In this paper the results are presented concisely.

## Note on subgeneric taxonomy

*Desmoceras* (*Desmoceras*) shows a depressed and subquadrate whorl section. The subgenus *Desmoceras* (*Pseudouhligella*) Matsumoto, 19383)–5) has been used for the species with oval whorl section and biconcave constrictions and lirae in the middle to late growth stages. These two subgenera have been generally approved, as shown by Wright.6) In this paper they may be indicated as *Desmoceras* (*D*.) and *Desmoceras* (*P.*) or *D.* (*D.*) and *D.* (*P.*) for brevity.

The shell surface of *Desmoceras* is not completely smooth. In the favorably preserved specimens, very weak and fine lirae or subcostae are discernible in every species in addition to the periodic constrictions and associated flares.

### 1. *Desmoceras (D.) latidorsatum (Michelin).*

In addition to the specimen reported by Matsumoto, 19545) (p. 248, pl. 6, fig. 5) from the Shuyubari area, we have treated two specimens from the lower part of the Upper Albian Member My1c of the Lower Yezo Group on the Sounnai route of the Soeushinai area: GK. H8497 and M. Hayashi’s Coll. no. 41 (now kept in the Nakagawa Museum). The former was contained in a large nodule, together with *Hysteroceras orbignyi* Spath etc.

### **2.** *Desmoceras ****(****P****.)*** *dawsoni shikokuense* (Yabe and Shimizu)

The holotype, IGPS.35154, was illustrated by Yabe, 19277) (pl. 3, fig. 10), without description. It is nomenclaturally approved by the description by Yabe and Shimizu (*in* Shimizu, 1931,8) p. 26, pl. 4, figs. 5, 6) under *Beudanticeras shikokuense*. On account of the difference in sutural pattern, this generic assignment was questionable.

**Fig. 2:**
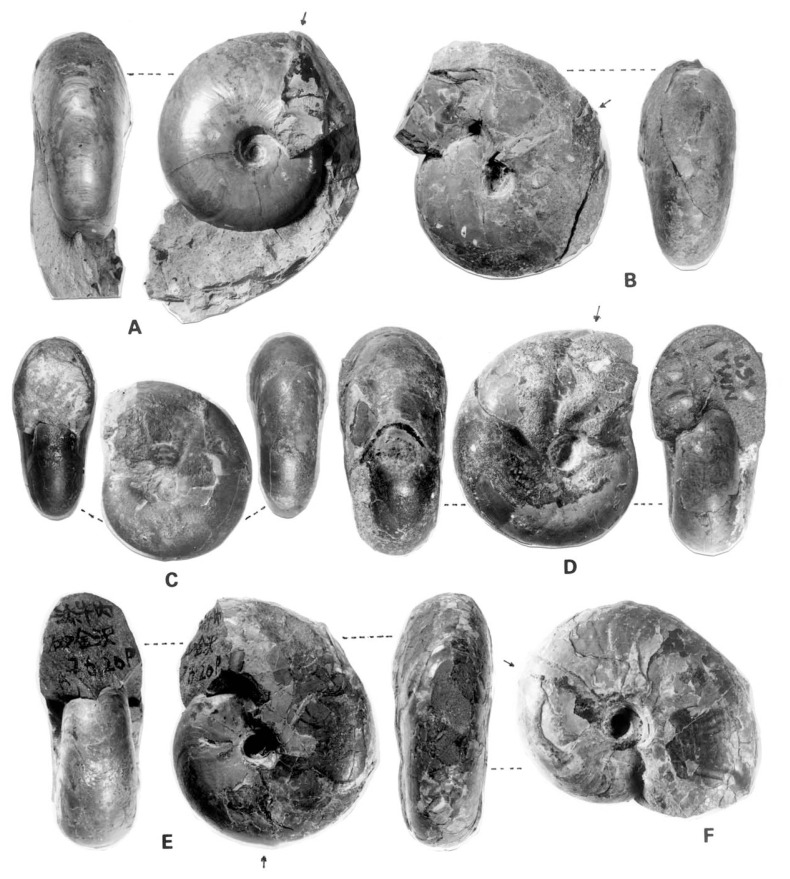
Selected examples of *Desmoceras*. A–C. *D*. *kossmati* Matsumoto. A: MCM. A345 from Hobetsu, collected by K. Hasegawa at a point 750 m east from the mouth of the creek called the Penke-wakka-tannenai-zawa. B: GK. H8625 from Loc. R8925p, Kyoei-Sakin-zawa of the Soeushinai area. C: GK. H8624, a young specimen, magnified × 2, from Loc. R519p2, eastern main branch of the Suribachi-zawa, a tributary of the Sounnai River. D: *Desmoceras japonicum* Yabe. NM. A157, middle-aged specimen from the Teshio Nakagawa, described by Hayakawa and Nishino, 1999.12) E–F: *Desmoceras japonicum minor* subsp. nov., GS. G097 from the Kyoei-Sakin-zawa, F is photoed under different lighting to show the weak subcostae or lirae. Figures are natural size, except C. Photos courtesy of M. Noda. In each figure an arrow indicates the position of the last septum. Abbreviations of the repositories: GK. = Geological Collection, the Kyushu University Museum, 6-10-1, Hakozaki, Higashi-ku, Fukuoka 812-8581, Japan; GS. = Geological Collection, Saga University, Honjo-machi, Saga 840-8502, Japan; MCM. = Mikasa City Museum, 1-212-1, Nishikicho, Ikushunbetsu, Mikasa, Hokkaido 068-2111, Japan; NM. = Nakagawa Museum of Natural History, 28-9, Yasukawa, Nakagawa-machi, Hokkaido 098-2626, Japan.

Much later, Nakai and Matsumoto (1968)9) gave a revised description on the basis of numerous specimens, including the holotype, from the Upper Albian Fujikawa Formation of Shikoku under *D*. (*P*.) *dawsoni shikokuense*. It is distinguished from *D*. *dawsoni dawsoni* (Whiteaves, 1900)10) by the smaller size of the adult shell and less flexuosity of the constrictions. In both subspecies the whorl section is narrowly subelliptical with a rounded umbilical margin. Its suture is well shown in some specimens from Shikoku (see Nakai and Matsumoto, 1968,9) fig. 2). This subspecies seems to occur also in the Upper Albian strata of Hokkaido, but the available specimens are secondarily distorted. We do not agree with Kawabe and Haggart11) in their assignment of this subspecies to *D*. (*P*.) *poronaicum*.

### **3.** *Desmoceras ****(****P****.)**** kossmati* Matsumoto.4),5)

The lectotype (UMUT MM6667 = GT. I-2551) was collected at Loc. N507p, Imano-sawa of the Naibuchi [= Naiba] area, S. Sakhalin. Its original horizon is at about the Albian–Cenomanian transition. In Hokkaido numerous specimens have been obtained from the Lower Cenomanian strata of the Soeushinai and Shuyubari areas, and also some from the uppermost part of the Albian in the same areas.

This species is characterized by the oval whorl section, which is compressed in youth and broadens with growth, resulting the whorl of later growth stage nearly as broad as high in some individuals. The shell size is generally small, e.g. about 40 mm diameter at the end of the phragmocone. The umbilical shoulder appears to be angular or subangular or even subrounded, falling into vertical wall. Constrictions occur rather infrequently and are better marked on the outer part of the whorl. They show forward biconcave curvature, although the concavity at the umbilical margin is less distinct.

To show the change of shell characters with growth and also some extent of variation, selected examples are illustrated in Fig. 2A–C in addition to the previous ones.5)

### **4.** *Desmoceras ****(****D****.)*** *japonicum* Yabe.1)

**Fig. 3:**
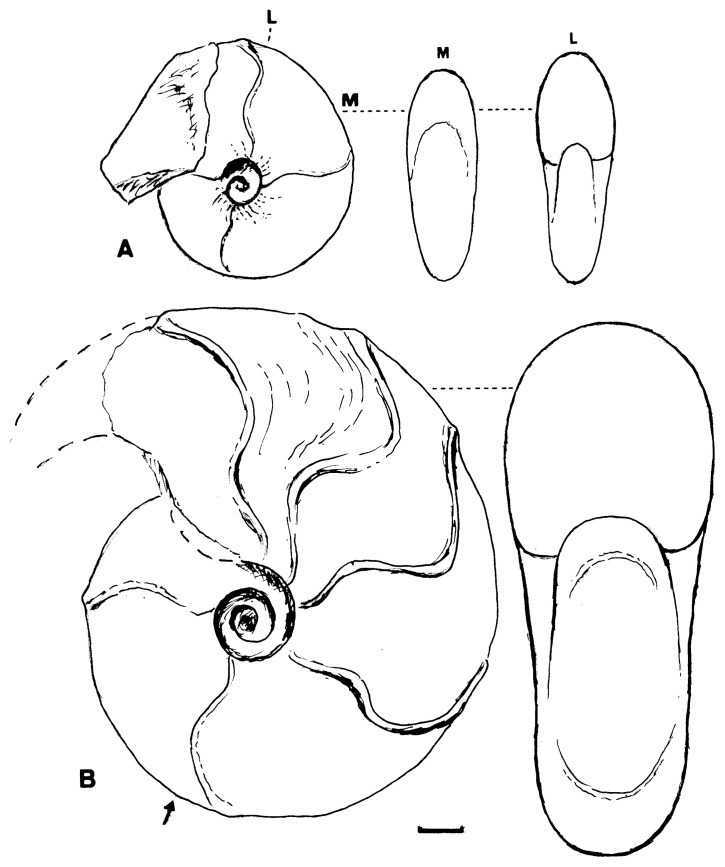
Diagrammatic figures of the holotypes of A: *D*. *ezoanum* Matsumoto and B: *D*. *japonicum* Yabe. Scale bar: 10 mm; arrow: end of phragmocone. A is wholly septate. Figures depend on the photos in Matsumoto, 1954,5) pl. 3 [19], fig. 1a–c and pl. 1 [17], fig. 7a, b.

This was described originally as *Desmoceras dawsoni* Whiteaves var. *japonica* Yabe1) (1904, p. 35, pl. 5, figs. 3, 4) and later ranked as a distinct species.4) Its holotype, UMUT MM7574 from Mikasa (Fig. 3B), is a nearly full grown shell, about 120 mm diameter, but its very apertural margin is destroyed. Some of the specimens from the Soeushinai area exhibit the apertural part, which has a remarkably forward projected venter and a highly sigmoidal margin of the flank, with well marked constrictions and accompanied flares behind. However, whether there is dimorphic (or sexual) difference in the apertural character or not is yet undecided. Moreover, whether a similar feature is maintained in other species of *Desmoceras* or otherwise has to be investigated further.

Except for the globular shape in very young stage (see Matsumoto, 1954,5) figs. 5, 6), the shell form of young stage (Fig. 2D) is essentially similar to that of the adult one. The whorl is nearly parallel-sided and the venter is evenly rounded. The umbilical wall is perpendicular to the plane of coiling, while the umbilical shoulder appears to be angular or subangular or even subrounded in some specimens.

The wide morphological variation of this species is observed in the material from various parts of the range of this species. For instance, at Loc. R8061 on the middle course of the Shumarinai River (Lower Cenomanian), a number of small shells (less than 25 mm in diameter) are contained in each of several nodules. They show variable breadth/height ratio even in one and the same nodule.

The difference in the shell size at maturity has been noticed between the specimens from the lower and upper stratigraphic levels. For some reasons small immature shells occur frequently in some cases. Through the intensive field work, we have noticed that young shells occur abundantly in some areas (e.g., the Soeushinai area), while mature shells occur frequently in some other areas (e.g., the upper reaches of the Shirakin-zawa in a part of the Yubari Mountains). Such difference may be related to the environmental conditions. Keeping this point in mind, we have examined various cases, and, thus we have arrived at the conclusion that even at the late growth stage the specimens from the lower stratigraphic levels (upper Albian and lower Cenomanian) are generally smaller than those from the higher levels (mainly middle part of the Cenomanian Stage). Thus, the subspecies can be proposed as follows: ***D*. (*P*.) *japonicum japonicum*** Yabe, 1904 as it is; and ***D*. (*P*.) *japonicum minor*** subsp. nov. GS. G097 (Fig. 2E–F) is here designated as the holotype of the latter. It came from the Lower Cenomanian Member My3 of the Kyoei-Sakin-zawa. It is 43 mm in diameter at the end of the phagmocone and its preserved part of the body chamber is about a half whorl.

The holotype of *D*. *poronaicum* Yabe, 19041) (p. 39, pl. 6, figs. 1, 2) is too small to show clearly the specific characters. However, it is nearly parallel sided, as shown by the photos in Matsumoto5) (1954, pl. 2 [10], fig. 5a,b). Its obliquely cut apertural view by Kawabe and Haggart11) (2003, fig. 6-1, -2) may give an erroneous image. Its record of occurrence by Yabe and the mode of preservation indicate its derivation from the particular fossiliferous layer in the southern part of the Poronai [recently called Horonai] area of Mikasa, where *Calycoceras* (*Newboldiceras*) *asiaticum* occurs. One of us (T. M.) visited there under the guidance of T. Takahashi, who is well acquainted with the fossiliferous outcrop. Based on this field evidence and observable characters of the specimens, *D*. *poronaicum* should be regarded as a junior synonym of *D*. *japonicum*. Some specimens which were erroneously referred to *D*. *poronaicum* by Matsumoto5) (1954, pl. 2 [18], figs. 6, 7; pl. 3 [19], fig. 7) should be transferred to *D*. *kossmati*.

Incidentally, the forms called *D*. (*P*.) *japonicum mediocompressa* and *D*. (*P*.) *japonicum compressior* by Matsumoto5) (1954, pp. 257–258) are merely varieties in *D*. (*P*.) *japonicum* and these subspecific names are unnecessary.

### **5.** *Desmoceras ezoanum* Matsumoto

The holotype, UMUT MM6705 (see Fig. 3A in this paper), came from Loc. T8431 of the Nakagawa district. This species ranges from the middle part of the Cenomanian to the lower part of the Turonian Stage in this and other areas of Hokkaido. It is characterized by a narrowly oval whorl section, with nearly flat to slightly convex flanks, converging to the narrowly arched venter. The umbilicus is narrow and surrounded by an angular or subangular shoulder. Its adult shell is fairly large. Its original description 4),5) is quite adequate.

## Concluding remarks

The above is the concise summary of our restudy of the *Desmoceras* from Japan and S. Sakhalin. It is different in some respects from the result of the recent study by Kawabe and Haggart (2003),11) who seem to have neglected the characters and stratigraphic position of the two original specimens of *D*. *poronaicum* Yabe.
